# Enhancement of Bone Marrow-Derived Mesenchymal Stem Cell Osteogenesis and New Bone Formation in Rats by Obtusilactone A

**DOI:** 10.3390/ijms18112422

**Published:** 2017-11-15

**Authors:** Yi-Hsiung Lin, Chung-Yi Chen, Liang-Yin Chou, Chung-Hwan Chen, Lin Kang, Chau-Zen Wang

**Affiliations:** 1 Orthopaedic Research Center, Kaohsiung Medical University, Kaohsiung 807, Taiwan; caminolin@gmail.com (Y.-H.L.); laining59@gmail.com (L.-Y.C.); hwan@cc.kmu.edu.tw (C.-H.C.); 2 Department of Physiology, College of Medicine, Kaohsiung Medical University, Kaohsiung 807, Taiwan; 3 Graduate Institute of Medicine, College of Medicine, Kaohsiung Medical University, Kaohsiung 807, Taiwan; 4 School of Medical and Health Sciences, Fooyin University, Kaohsiung 807, Taiwan; XX377@fy.edu.tw; 5 Department of Orthopaedics, College of Medicine, Kaohsiung Medical University, Kaohsiung 807, Taiwan; 6 Department of Orthopedics, Kaohsiung Municipal Ta-Tung Hospital, Kaohsiung Medical University, Kaohsiung 807, Taiwan; 7 Division of Adult Reconstruction Surgery, Department of Orthopedics, Kaohsiung Medical University Hospital, Kaohsiung Medical University, Kaohsiung 807, Taiwan; 8 Department of Obstetrics and Gynecology, National Cheng Kung University Hospital, College of Medicine, National Cheng Kung University, Tainan 701, Taiwan; kanglin@mail.ncku.edu.tw; 9 Department of Medical Research, Kaohsiung Medical University Hospital, Kaohsiung 807, Taiwan

**Keywords:** bone marrow derived mesenchymal stem cells (BMSCs), osteogenesis, bone formation, *Cinnamomum kotoense*, obtusilactone A

## Abstract

The natural pure compound obtusilactone A (OA) was identified in *Cinnamomum kotoense* Kanehira & Sasaki, and shows effective anti-cancer activity. We studied the effect of OA on osteogenesis of bone marrow-derived mesenchymal stem cells (BMSCs). OA possesses biocompatibility, stimulates Alkaline Phosphatase (ALP) activity and facilitates mineralization of BMSCs. Expression of osteogenesis markers *BMP2*, *Runx2*, *Collagen I*, and *Osteocalcin* was enhanced in OA-treated BMSCs. An in vivo rat model with local administration of OA via needle implantation to bone marrow-residing BMSCs revealed that OA increased the new bone formation and trabecular bone volume in tibias. Micro-CT images and H&E staining showed more trabecular bone at the needle-implanted site in the OA group than the normal saline group. Thus, OA confers an osteoinductive effect on BMSCs via induction of osteogenic marker gene expression, such as BMP2 and Runx2 expression and subsequently elevates ALP activity and mineralization, followed by enhanced trabecular bone formation in rat tibias. Therefore, OA is a potential osteoinductive drug to stimulate new bone formation by BMSCs.

## 1. Introduction

*Cinnamomum kotoense* Kanehira & Sasaki is a small evergreen tree endemic to Lanyu Island, Taiwan, is used as a dietary supplement, and its constituents exhibit various bioactivities, such as anti-proliferation and anti-tumoral activities against HeLa cells [1]. The pure compounds isokotomolide A, secokotomolide A, obtusilactone A (OA) and (-)-sesamine have been extracted from *C. kotoense* recently. It was found that OA and (-)-sesamine can induce DNA damage and lead to cell cycle arrest in the G1/S-phase, followed by cell death of lung cancer by inhibiting human Lon protein expression [1,2]. We recently reported that OA exerts effective radical scavenging activity that is comparable to vitamin C [3]. However, the osteoinductive effect of the pure compounds of *C. kotoense*, including OA, on osteogenic cells remains undefined.

Bone marrow-derived mesenchymal stem cells (BMSCs) are multipotent stromal cells that can differentiate into a variety of cell types including osteoblasts, chondrocytes, myocytes and adipocytes [4]. The differentiation of BMSCs into osteogenic/osteoprogenitor cells is a promising therapeutic method to promote new bone formation and heal fractures. The direct application of BMSCs in open surgery has been reported with encouraging results in patients with delayed bone fracture healing [5]. BMSC-based bone tissue engineering with effective osteoinductive factors are necessary for enhancing new bone formation and bone regeneration [5,6,7].

Various natural compounds have broad effects toward various diseases, and their therapeutic mechanisms may differ. Recently, studies have shown that the application of natural compounds, including Taxol, curcumin and paclitaxel, promote the cure of cancer, inflammation, and many other diseases [8,9,10]. Growth factors, such as BMP2, have potent effects on BMSCs for bone development and new bone formation [11]. However, drawbacks of growth factors include their easy degradation and high cost. In contrast, natural pure compounds are relatively stable and easily accessible and, therefore, are worth screening for their osteoinductive effects in applications of BMSC-based bone tissue engineering. However, few studies have considered the osteoinductive effects of natural pure compounds on BMSCs. Herein, we investigated the effects of the natural pure compound OA on stimulating BMSC osteogenesis for new bone formation, and the possible molecular mechanisms of induction of BMSC osteogenesis were determined.

## 2. Results

### 2.1. Determination of the Bioactivity Effects of Purified Compounds of Cinnamomum kotoense on BMSCs

**Figure 1:**
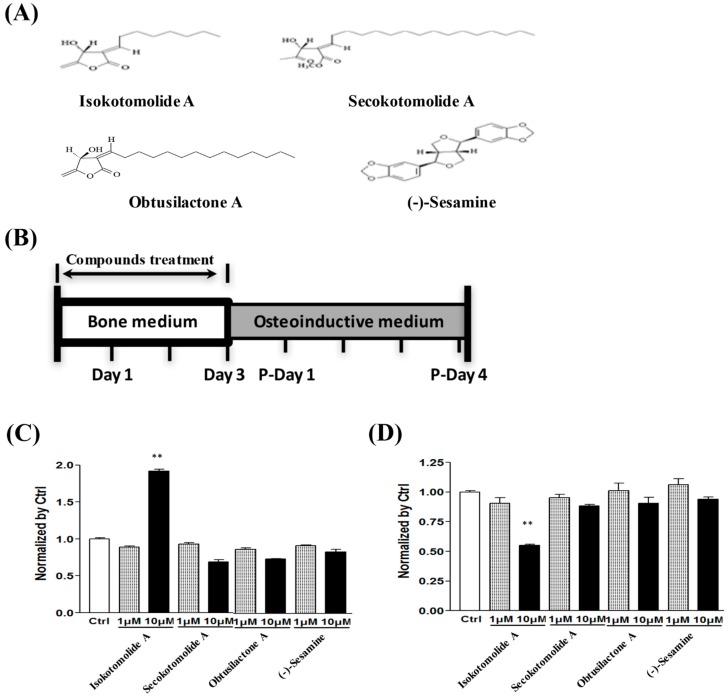
Determining the cytotoxic effects of pure compounds on BMSCs: (**A**) structures of the extracted pure compounds from the leaves of *Cinnamomum kotoense*; (**B**) treatment time-line for analyzing the osteoinductive effects of extracted pure compounds from *C. kotoense* on BMSCs, P-Day: day post OIM replacement; (**C**) cytotoxicity analysis of BMSCs after treatment with 0.01% DMSO as a control group or a variety of pure compounds at concentrations of 1 μM and 10 μM for one day via LDH assays; and (**D**) MTT assays were used to determine cell viability after treatment with pure compounds extracted from *C. kotoense* for three days. ** *p* < 0.01.

We first determined the cytotoxic effect of the pure compounds of *Cinnamomum kotoense* Kanehira & Sasaki via an LDH leakage test. After 24 h incubation with the indicated compounds and concentrations in normal bone medium, we found that 10 μM isokotomolide A induced intense cytotoxic effects on BMSCs (Figure 1C). In addition, cell viability analysis based on the MTS assay revealed 50% BMSC viability after isokotomolide A treatment for three days (Figure 1D). In contrast, the other drugs (10 μM), including secokotomolide A, OA and (-)-sesamine, exhibited no significant cytotoxic effect (Figure 1C) on BMSCs, and cell viability (Figure 1D) was similar to the control group. As isokotomolide A induced intense cytotoxicity on BMSCs, it is not suitable as an osteoinductive drug.

### 2.2. Osteoinductive Effect of Pure Cinnamomum kotoense Compounds on BMSCs Osteogenesis

**Figure 2:**
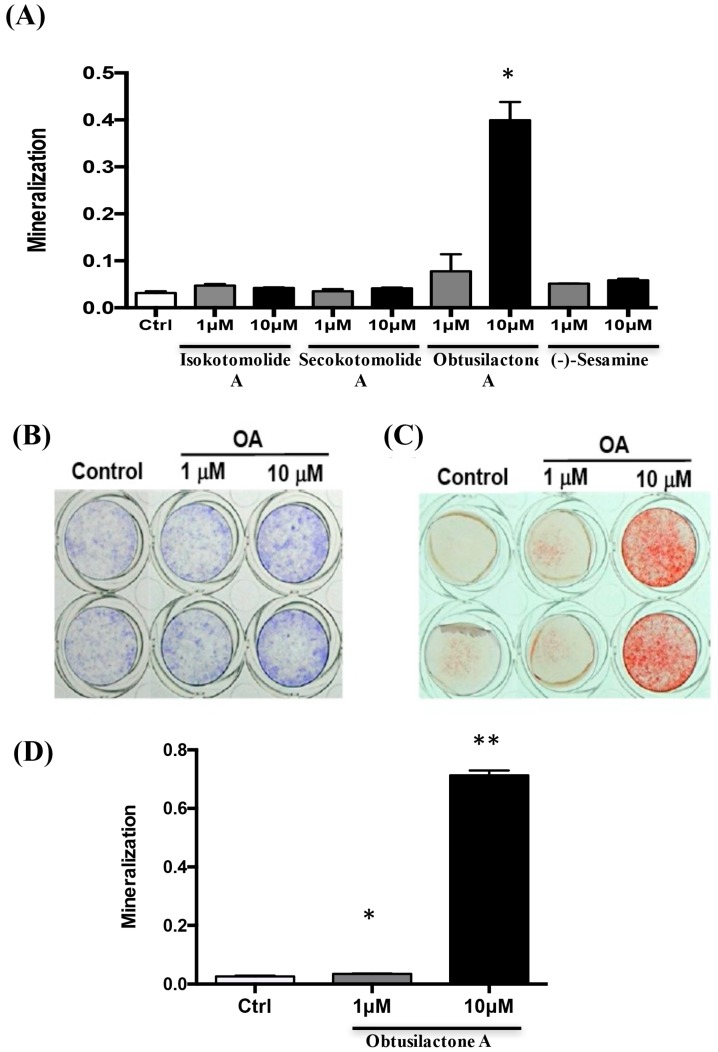
Obtusilactone A induced osteogenic differentiation of BMSCs: (**A**) pre-screening of the mineralization stimulatory ability of the natural pure compounds extracted from *C. kotoense* in BMSCs based on a mineralization assay; (**B**) determination of the ALP activity of BMSCs after treatment with 0.01% DMSO (control) or 1 and 10 μM OA for three days; (**C**) mineralization assay of BMSCs after OA treatment; and (**D**) quantified results of the mineralization assay. Data are presented as the mean of three independent experiments. The data shown are the means ± SD of 3 independent experiments. * *p* <0.05, ** *p* <0.01 for compounds-treated versus control groups.

In studies of osteogenic effects in vitro, mineralization is considered an in vitro endpoint that reflects osteogenic differentiation. We next prescreened the osteoinductive effect of pure compounds of *Cinnamomum kotoense* on BMSC osteogenesis via a mineralization assay. BMSCs were treated with 1 or 10 μM pure compound for three days in normal bone medium, followed by replenishing the medium with osteoinduction medium for osteogenic differentiation of BMSCs without any compounds. As shown in Figure 2A, 10 μM OA induced the highest degree of mineralization among the compounds after 96-h incubation in osteoinductive medium. Considering the bioavailability and osteoinductive potential of these pure compounds of *Cinnamomum kotoense*, obtusilactone A (OA) was selected as the candidate compound for further investigation. After three days of OA treatment and osteoinductive medium replacement, an Alkaline Phosphatase (ALP) activity assay and Alizarin Red S (ARS) staining were performed at 24 and 96 h post medium refresh, respectively, to confirm the ability of OA to stimulate BMSC osteogenesis. The ALP activity assay showed that OA-treated BMSCs had increased ALP activity compared with control cells (Figure 2B). The mineralization assay showed that 10 μM OA induced a significant stimulatory effect on mineralization (Figure 2C) with over 25-fold increased mineralization compared with the control group and 1 μM OA group (Figure 2D). These data indicated that OA possesses osteoinductive potential for inducing osteogenesis of BMSCs.

### 2.3. Enhancement of Osteogenic Gene Expression of BMSCs after OA Treatment

**Figure 3:**
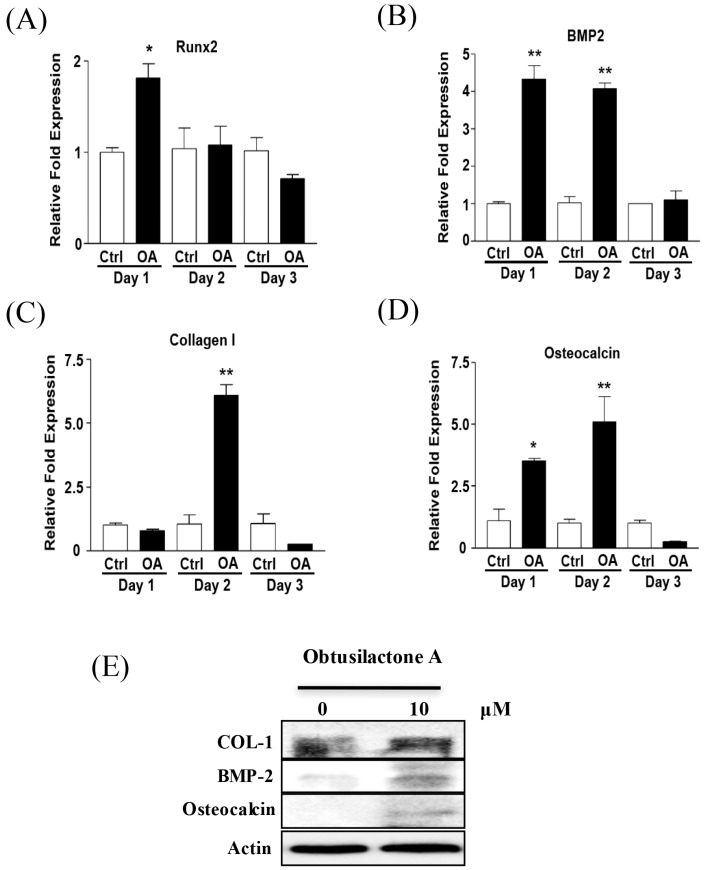
OA enhances osteogenic marker gene expression in BMSCs. The osteogenic marker gene expression levels of: (**A**) *Runx2*; (**B**) *BMP2*; (**C**) *Collagen I*; and (**D**) *Osteocalcin* in OA treated-BMSCs were examined by Q-PCR analysis. (**E**) Western blot of the protein expression of BMP2, Collagen I, Osteocalcin and β-actin (internal control) in BMSCs after treatment with 10 μM OA for 2 days. * *p* < 0.05 and ** *p* < 0.01.

We next investigated the osteoinductive potential of OA on the expression of osteogenic marker genes, including *Runx2*, *BMP2*, *Collagen I* and *osteocalcin*, during BMSC osteogenesis using qRT-PCR after treatment with OA for three days. We found that *Runx2* (Figure 3A), *BMP2* (Figure 3B) and *osteocalcin* (Figure 3D) were up-regulated by 2–4-fold compared to the control group (0.01% DMSO) one day after OA treatment. The mRNA expression levels of *BMP2* and *osteocalcin* were continuously expressed (4 and 5-fold of control respectively) after two days of treatment and restored to baseline after three days of OA treatment. *Type I collagen* was up-regulated by over 6-fold of control after two days of treatment and returned to normal after three days of OA treatment (Figure 3C). However, the gene expression of other osteogenic marker genes including *bone sialoprotein (BSP)*, *osterix* and *osteopontin* were not different between control and OA groups (data not shown). In addition, there was also no difference in the expression of chondrogenic marker genes (*Sox-9, Col II* and *aggrecan*) and adipogenic marker genes (*peroxisome proliferator-activated receptor γ (PPARγ)*, and *CCAAT/enhancer binding proteins (C/EBPs) α, β* and *δ*) between control and OA-treated BMSCs (data not shown). Because the expression of osteogenic marker genes was elevated the greatest at Day 2 after OA treatment, Western blot was performed to analyze the protein expression of these osteogenic marker genes at Day 2 after OA treatment. The results showed that BMP2 and osteocalcin markedly increased, and type I collagen slightly increased after two days of OA treatment (Figure 3E). These results revealed the osteoinductive mechanisms of OA through enhancing osteogenic marker gene expression to induced BMSC osteogenesis.

### 2.4. Local Administration of OA Enhanced New Bone Formation in the Tibia in Rats

**Figure 4:**
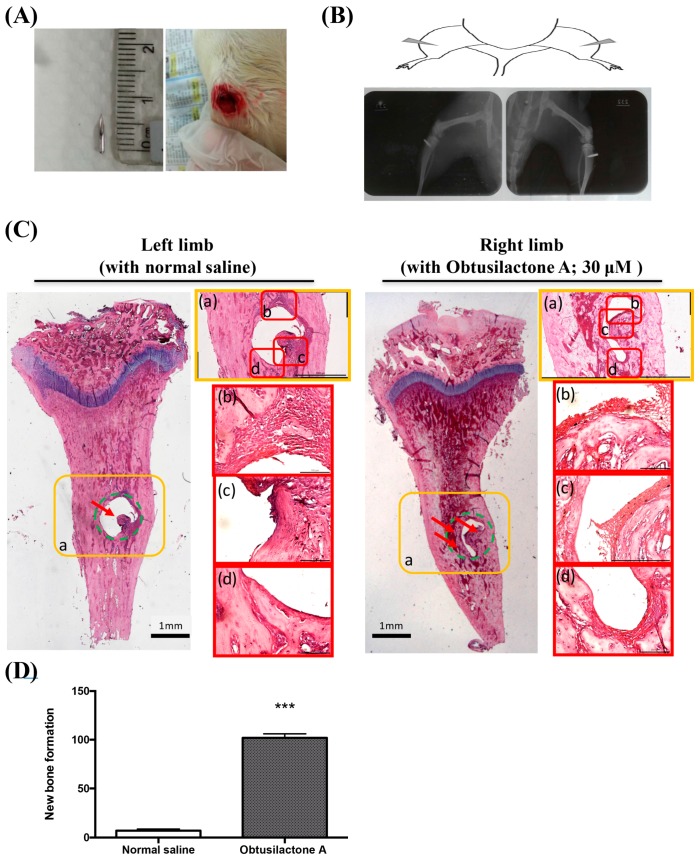
Evaluation of new bone formation via needle implantation of OA into the proximal tibial metaphysis of rats. OA (30 μM, 30 μL, once/two day) was locally administered into the tibia via a needle. (**A**) The cut tip of the needle (4 mm) and the posteriolateral side of the proximal tibial metaphysis in both hindlimbs. (**B**) Schematic view and X-ray film of the needle implantation side. (**C**) H&E staining of the needle implantation site in the OA-treated right tibia (right panel; enlarged **a**–**d**) and normal saline-treated left tibia (left panel; enlarged **a**–**d**) in rats after 14 days administration (green dotted line: the original size of the needle defect; red arrow: the new bone formation). (**D**) Quantified analysis of new bone formation inside the region of interest we selected as the original bone defect. *** *p* < 0.005.

To evaluate the osteoinductive and new bone formation effects of OA on BMSCs in vivo, needle implantation of OA into rat tibias was performed to evaluate the effect of local administration of OA on stimulating BMSCs that reside in the bone marrow of the tibia. Twenty-gauge needles (4 mm; Figure 4A) were implanted in the tibias of rats (Figure 4B) for local administration of normal saline (30 μL, once/two days) or OA (30 μM, 30 μL, once/two days). After 14 days of injection, the tibias were collected for micro-CT scanning and paraffin sectioning for Hematoxylin and Eosin (H&E) staining. X-ray images indicated the needle implantation sites in both tibias (Figure 4B). H&E staining at needle implanting site showed more new bone formation in the OA-treated tibias than in the contralateral normal saline-treated tibias inside the original defect site (green dotted circle; Figure 4C; enlarge a–d). It was found that the dramatically increased of new bone formation observed in the OA-treated tibias (red arrow; Figure 4C). The quantified result of H&E staining image in defect site also showed that OA induced over 15 folds new bone formation compared with control saline treatment group (Figure 4D).

**Figure 5:**
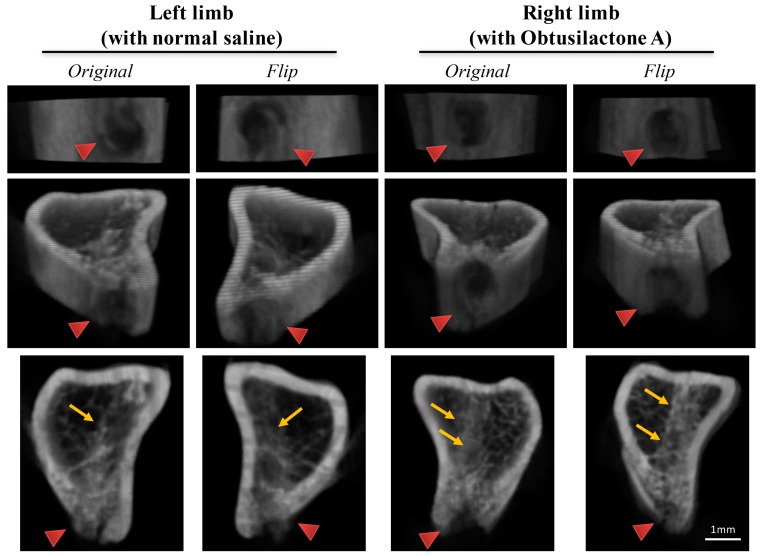
3D reconstructed micro-CT images show enhanced trabecula bone formation in OA-treated rat tibias. The original and flip 3D micro-CT images around the ROI of the tibia (4-mm radius around 1.0-mm up and down from the region of the needle implantation site; 100 cuts) in OA-treated right tibias (right panel) and normal saline-treated left tibias (left panel) of rats after 14 days of administration (red arrow head: the needle implantation site; yellow arrow: the new-formed trabeculae).

The observation was confirmed and quantified by Micro-CT reconstruction analysis. As shown in Figure 5, the original and flip images of the 3D reconstructed micro-CT results revealed that the beginning defect site (red arrow; Figure 5) and the new-formed trabeculae (yellow arrow; Figure 5). The results showed that the OA-treated tibias exhibited more trabecular formation in the bone marrow of the injection site than contralateral saline-treated tibias.

**Figure 6:**
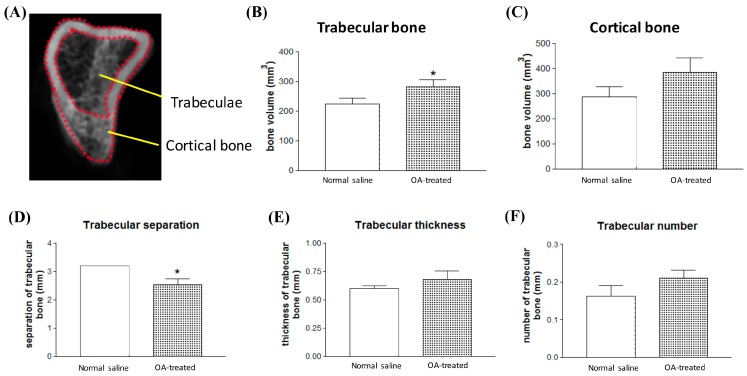
The 3D morphometric parameters show increased trabecular bone volume in OA-treated rat tibias. (**A**) Defined ROI for the trabeculae volume (inside the red dotted line) and cortical (between the red dotted line) bone volume analysis. (**B**) Cortical bone; and (**C**) trabecular bone volume measurement using micro-CT analysis CTAn software. Bone mass index of trabecular bone evaluation including: (**D**) trabecular separation (Tb.Sp).; (**E**) trabecular thickness (Tb.Th).; and (**F**) trabecular number (Tb.N). * *p* < 0.05.

To further evaluate the new bone formation between normal saline-treated and contralateral obtusilactone A-treated tibia, the region of interest (ROI) of compact bone and cancellous bone were selected and defined for 3D Micro-CT images reconstruction and bone volume quantitative analysis (Figure 6A). In OA-treated tibia, trabecular bone volume significantly increased by over 30% (Figure 6C), but no significant difference in cortical bone volume was observed (Figure 6B). Trabecular bone indices, including trabecular thickness (Tb.Th), trabecular separation (Tb.Sp) and trabecular number (Tb.N), are important parameters of the 3D microstructure of trabecular bone. In OA-treated tibia, trabecular separation was significantly decreased by approximately 20% compared with contralateral saline-treated tibia (Figure 6D), and had the trend to increase but without significace in the trabecular thickness (Figure 6E) and trabecular number (Figure 6F). These results suggest that increased trabecular bone volume in OA-treated tibia may via increase trabecular thickness and trabecular number, which decreased the trabecular separation in OA-treated tibia. These findings indicate that OA exhibited osteoinductive effects for inducing BMSC osteogenesis for trabeculae-particular new bone formation.

## 3. Discussion

Natural products, such as carvacrol, Bajijiasu and Monotropein, have been previously reported to regulate RANKL signaling and NF-κB in osteoclastogenesis and osteoclasts to prevent bone loss [12,13]. The natural pure compound OA extracted from edible plants *Cinnamomum kotoense* was reported to exhibit many pharmacological properties, including anti-cancer, anti-inflammatory and antioxidant effects and free radical-scavenging activities. In this study, we demonstrated that OA exhibited potent osteoinductive effects for inducing BMSC osteogenesis and new bone formation. We found that OA induced osteogenesis in BMSCs by stimulating ALP activity and mineralization. Most importantly, OA exhibited good biocompatibility without inducing cytotoxicity or inhibiting cell proliferation of BMSCs. Several studies have reported that Runx2 and BMP2 are useful osteogenic markers of osteogenesis and play roles in regulating bone mineral density (BMD) [14,15]. In addition, BMP2 can activate the transcription factor Runx2 [16,17]. It has also been reported that TGF β/BMP signaling will regulate the *Runx2* gene promoter through different Smads subfamily, such as Smad 2/3 and 4 phosphorylation and Smad 7 ubiquitination. Then, the active complex translocates into nuclear and binds to SBE transcription receptor of *Runx2* promoter [18]. Our in vitro results support this previous finding and showed that OA stimulated new bone formation through increasing osteogenic marker genes including *BMP2*, *Runx2*, *Collagen I* and *Osteocalcin*. OA increased the *Runx2* expression in transcriptional level, and this activation could be possibly contributed by both TGF β and BMP2 superfamily, while we only determined the expression of BMP2. BMP7 is a well-known osteogenesis inducer that promotes osteogenic differentiation as well, and seems to activate through the different pathway of BMP2. It has been reported that the two pathways should have cross-talk while they induce osteogenic differentiation by triggering each other [19]. Accordingly, OA induced osteogenesis and BMP2 and Collagen I expression in BMSCs could also accompany with the BMP7 activation, which all contributed to new bone formation finally. Further supporting our in vitro results, we showed that OA exhibited the ability to stimulate osteogenesis of BMSCs in bone marrow and to increase new bone formation of trabecular bone in rat tibias.

According to previous cytotoxic results of pure compounds of *Cinnamomum kotoense* [1,2], in this study, to minimize their cytotoxic effect on BMSCs, the dosage of pure compounds for evaluating cytotoxicity, the following osteogenic effects on BMSCs were tested at concentrations up to 10 μM. Although the cytotoxic effect of OA on osteoblasts and osteoclasts was not investigated in this study, the in vitro results of MTS and LDH assays both showed that OA induced extremely low cytotoxicity on BMSCs. Most importantly, through H&E stain image, we found that local administration of OA to the bone marrow of rat tibias induced no obvious inflammation with neutrophil stained in the tibias or bone marrow and promoted a significant increase in trabecular bone volume. According to these findings, we speculate that the dosage of OA we used in this study may induce only a little cytotoxic effect on osteoblasts and osteoclasts. However, further studies are needed to characterize the detailed effects of OA on osteoblasts and osteoclasts and to clarify the effects of local administration of higher doses or longer treatment periods of OA in vivo.

Despite the clinical need for therapeutics aiming to increase bone formation of BMSCs, effective methods to evaluate potential osteoinductive drugs on inducing BMSC osteogenesis are urgently needed. The animal model used in the present study provides an easy and effective way to evaluate the efficiency of osteoinductive drugs in vivo. We used a needle implantation model to evaluate the bioavailability and the osteoinductive effect of OA on BMSCs inside the bone marrow of rats’ tibias. The injection volume of OA (30 μM) was reduced to 30 μL for local administration to imitate the final concentration of approximately 10 μM OA in the bone marrow of rat tibias. Using this model, we could inject OA directly and locally to determine its inductive effect on new bone formation by using micro-CT analysis and H&E staining, which showed that new bone formation in OA-treated tibias was better than that of contralateral saline-treated tibias. By implanting a needle in both legs for treatment with OA and normal saline in the same rat, we obtained results with less individual differences for comparison. Although OA may have some interference on new bone formation in the contralateral tibia, our animal model demonstrated that OA has potent osteoinductive effects on BMSCs inside the bone marrow of rats’ tibias. Moreover, the in vivo results were consistent with our in vitro findings that OA could stimulate the osteogenesis of BMSCs, and it was double confirmed by quantified analysis of H&E staining and Micro-CT images. Taken together, our animal model can be used for evaluating the in vivo osteoinductive potential of new drugs on BMSCs.

## 4. Materials and Methods

### 4.1. Bone Marrow-Derived Mesenchymal Stem Cell Preparation

BMSCs cloned from Balb/C mice were purchased from the American Type Culture Collection (ATCC). The BMSCs have multipotent differentiation capacity and were found to be primarily osteogenic, and they were able to “home” back to bone marrow and participate in fracture repair upon either systemic or local injection [20]. Cells were maintained in Dulbecco’s modified Eagle’s medium (DMEM) as normal bone medium with 10% fetal bovine serum (Gibco, BRL, Bethesda, MD, USA), 50 mg/mL sodium ascorbate, 50 units/mL penicillin and 50 μg/mL streptomycin (Gibco BRL, Bethesda, MD, USA). The cells were seeded at a density of 4 × 10^4^ cells per cm^2^ in a six-well plate and cultivated in a humidified atmosphere of 5% carbon dioxide at 37 °C. The media were then replaced with osteoinductive media (OIM) consisting of low-glucose DMEM, 2.2 mg/mL sodium bicarbonate, 10% fetal bovine serum, 100 nM dexamethasone, 10 mM b-glycerophosphate, 0.1 mM l-ascorbic acid-2-phosphate, and 0.5% penicillin (10,000 U/mL)/streptomycin (10,000 lg/mL) to induce osteogenic differentiation for additional 1 or 4 days. Experiments were performed after the cells reached approximately 80% confluence.

### 4.2. Natural Pure Compound Treatment

The natural pure compounds isokotomolide A, secokotomolide A, obtusilactone A (OA) and (-)-sesamine (Figure 1A) were extracted from the leaves of *Cinnamomum kotoense Kanehira* and the extracted methods were described previously [3]. The extraction powders of the natural pure compounds were dissolved in 0.01% DMSO at a concentration of 10 mM and kept at −20 °C for the remaining experiments. The compound stocks were diluted with culture medium immediately before treatment. Cells were treated with 0.01% DMSO as a control group or with variety of compounds at concentrations of 1 μM and 10 μM. The culture medium (prepared with DMEM, 10% FBS, 10 mM nonessential amino acids, 0.01% vitamin C, 50 units/mL penicillin and 50 μg/mL streptomycin) was changed every other day with the indicated concentration of these compounds. To examine mRNA expression of osteogenic markers, BMSCs were treated with OA for 3 days. For the alkaline phosphatase (ALP) activity assay, cells would be treated with the indicated concentration of compounds for 72 h before changing to osteo-inductive medium (OIM), and harvested 1 day post medium changing (Day 4). In the mineralization assay, the same treatment procedure of compounds and time-line before OIM and Alizarin Red S staining was performed 4 days post OIM changing (Day 7) (Figure 1B). The experiments were repeated at least 3 times for *t*-test statistical analysis.

### 4.3. MTS Cell Proliferation Assay

Cell viability was quantitatively assessed with the tetrazolium compound, 3-(4,5-dimethylthiazol-2-yl)-5-(3-carboxymethoxyphenyl)-2-(4-sulfophenyl)-2H-tetrazolium, inner salt (MTS; Sigma, Darmstadt, Germany). To evaluate the effect of these natural compounds on BMSC cell viability, 5000 BMSCs per well were seeded on a 96-well cell culture plate in culture medium with 0.01% DMSO as a control group or with two different concentrations (1 and 10 μM) of the natural compounds for 3 days. Culture plates with the culture medium only were used as a blank control. After cultivation, 10 μL MTS solution (5 mg/mL in Phosphate Buffered Saline; PBS) was added to each well and incubated for another 4 h (37 °C). The optical density (O.D.) of the produced water-soluble formazan in the solution was measured with an ELISA reader (SLT, Crailheim, Germany). Data were collected and averaged from five different wells per condition. Data were plotted as the mean ± standard deviation.

### 4.4. LDH Cytotoxicity Assay

The cultured BMSCs were individually incubated with the indicated concentrations of natural compounds to induce cytotoxicity and to subsequently release LDH. We used an LDH Cytotoxicity Assay Kit (Thermo Sci, Rockford, IL, USA) to measure the release of LDH in the culture medium. LDH was released from the treated cells into the medium and was then transferred to a 96-well plate mixed with the reaction mixture. After 30 min of incubation at room temperature, a stop solution was added to end the reaction. LDH activity was determined by the absorbance at 490 nm using a plate-reading spectrophotometer. The promotion rate of mineralization was calculated according to the following formula: (OD_sample_ − OD_control_)/OD_control_ × 100.

### 4.5. Quantitative Real-Time PCR Analysis

Total RNA was separated with chloroform and precipitated with isopropyl alcohol after homogenizing with Trizol reagent (Gibco BRL, Bethesda, MD, USA). The RNA pellet was washed with 75% ethanol, and the RNA was redissolved in RNAse-free water. The concentration of RNA was quantified by measuring the absorbance at 260 and 280 nm with a spectrophotometer. A sample of 1 μg total RNA was reverse transcribed by Moloney murine leukemia virus RT and a random hexamer primer (Applied Biosystems, Branchburg, NJ, USA). Polymerase chain reaction (PCR) was performed with a Perkin-Elmer Gene Amp 9700 PCR system (Applied Biosystems, Branchburg, NJ, USA). The PCR reaction was carried out with specific primers for each gene and a thermostable DNA polymerase (Gibco BRL, Bethesda, MD, USA). According to our previous report, the osteogenic marker gene changes in mRNA expression levels of *BMP2*, *osteocalcin*, *type I collagen* and *Cbfa1/Runx2* were then analyzed [21,22]. The following mouse primer pairs were used: *Osteocalcin (OC)* (5′-CTTGGTGCACACCTAGCAGA-3′ forward and 5′-CTCCCTCATCGTGTT GTC CCT-3′ reverse) and *Cbfa1/Runx2* (5′-CGCTCCGCCCACAAATCTC-3′ forward and 5′-CCGCACGACAACCGCACCAT-3′ reverse), *BMP2* (5′-TGCGGTCTCCTAAAGGTCG-3′ forward and 5′-GAGGACCTGGGGAAGCA-3′ reverse), *Collagen I* (5′-GGGGCAAGACAGTCATCGAA-3′ forward and 5′-GGGTGGAGGGAGTTTACACG-3′ reverse) and *GAPDH* (5′-ATACGGCTACAGCAACAGGG-3′ forward and 5′-GCCTCTCTTGCTCAGTGTCC-3′ reverse) as a house-keeping gene.

### 4.6. Alkaline Phosphatase Activity Assay

ALP activity of cells reflects osteogenic cells undergoing terminal differentiation. To elevate the ALP activities, BMSCs were seeded at 3 × 10^4^ cells per cm^2^ in a six-well plate in culture medium. Cells were cultured for 4 days in medium with or without OA, and the medium was changed every other day (with the same concentration of indicated compounds). The culture medium were aspirated and wash with 1× PBST before fixation solution. After 2 min cell fixed with 500 μL for each well of 24-well, cell was rinsed with 1× PBST and should not be dry out. Staining solution was freshly prepared and used within five min of preparation. Cover the plate with aluminum foil in the dark for approximately 15 min and avoid non-specific staining. The staining solution were removed and replaced with 1× PBS. AP stained pluripotent colonies and the differentiated colonies were counted by eye using light microscope with 10× objective.

### 4.7. Mineralization Assay by Alizarin Red S Staining

A mineralization assay was performed by Alizarin Red S staining as previously reported [21]. Briefly, BMSCs were cultured with culture medium and incubated with/without the indicated concentrations (1 and 10 μM) of individual natural pure compounds for 3 days. The culture medium was then replaced with osteoinduction medium consisting of low-glucose DMEM, 2.2 mg/mL sodium bicarbonate, 10% fetal bovine serum, 100 nM dexamethasone, 10 mM β-glycerophosphate, 0.1 mM L-ascorbic acid-2-phosphate, and 0.5% penicillin (10,000 U/mL)/streptomycin (10,000 lg/mL) to induce mineralization. After 4 days of treatment, cells were washed twice with distilled water, fixed in ice-cold 70% (*v*/*v*) ethanol for 1 h, rinsed twice with deionized water, and stained with Alizarin Red S (Figure 1B). Alizarin Red S was prepared in deionized water and adjusted to pH 4.2. After staining for 10 min at room temperature, excessive dye was gently washed out with running water. Calcification deposits were stained red. Mineralization was quantified by using 100 mM cetylpyridinium chloride to extract the stained Alizarin Red S at room temperature for 3 h, and then, the absorbance of the extracted Alizarin Red S stain was measured at 570 nm.

### 4.8. Needle Implantation in the Tibias of Rats

Eight-week-old Sprague Dawley rats (SD) rats were purchased from the National Laboratory Center, and in vivo experiments were performed with the approval of the Kaohsiung Medical University Animal Care and Use Committee (approval code: 104118; date: 1 August 2016). To investigate the effects of OA on BMSC osteogenesis in vivo, a needle implantation experiment was performed to evaluate new bone formation with local administration of OA in the bone marrow of rat tibias. In brief, 20-gauge needles were cut to 4 mm and sterilized for implantation preparation. The needles were then implanted into both tibias of SD rats approximately 4 mm from the head of tibia for local injection of OA or normal saline. OA was dissolved in normal saline (30 μM, 30 μL) immediately prior to the experiments. A total of 8 rats were used for the needle implantation experiment. OA (30 μL, once/2 days) was locally administered into the right tibia of the rats, and normal saline (30 μL, once/2 days) was administered into the left tibia of the rats for comparison for 14 days. Afterward, the rats were sacrificed, and the tibias were harvested for the following analysis.

### 4.9. Hematoxylin and Eosin Staining and X-ray Radiological Photography of Rats’ Limbs

Harvested tibia were fixed with 10% formalin solution, demineralized at 4 °C using 0.34 M EDTA, embedded in paraffin and then sectioned at a thickness of 5 μm. Sections were histologically analyzed by staining sections with hematoxylin (H3136, Sigma) and eosin (318906, Sigma) (H&E) and observed via microscopy.

The accurate needle implantation site was confirmed by normal X-ray procedure: 13 cm × 13 cm images, based on a-Si technology with 143 μm × 143 μm pixel image size, were obtained using Fuji EC-MA cassette (Tokyo, Japan). Film focus distance was 80 cm and the picture was shot with exposure time 1.5 s and 40 kV in the posterior-anterior.

### 4.10. Micro-Computed Tomography and Bone Volume Evaluation

To characterize bone formation at the needle implantation site in more detail at the ultrastructural level, 3D reconstructions of the tibia were generated using a high-resolution micro-computed tomography (Micro-CT) analysis (Skyscan 1076; Skyscan NV, Kontich, Belgium). Tibias were harvested after local administration of OA for 14 days and then scanned at an isotropic voxel resolution of 9 μm without a filter with 55 kV X-ray tube voltage, 200 μA tube electric current, and 2000 ms scanning exposure time. 3D images were reconstructed for analysis using a scale of 0–0.09 (NRecon version 1.6.10.4; Skyscan NV, Kontich, Belgium). To evaluate the individual bone index of total, trabecular and cortical bone appropriately, the different selected area of the trabecular and cortical bone were clearly defined.

The 3D morphometric parameters were computed using a direct 3D approach (CTAn version 1.16.1.0) in the ROI of the tibia (4-mm radius around 1.0-mm up and down the region of the needle implantation site; 100 cuts), which included bone volume (BV, mm^3^), bone thickness (μm), trabecular thickness (Tb.Th, μm), trabecular separation (Tb.Sp, μm) and trabecular number (Tb.N, mm^−1^).

### 4.11. Statistical Analysis

Data were presented as the mean ± standard deviation. All data were evaluated by one-way analysis of variance (ANOVA), Scheffe’s method and non-parametric statistics Mann–Whitney analysis. *p* < 0.05 was considered significant.

## 5. Conclusions

In conclusion, our results indicate that can OA induce BMSC osteogenesis by enhancing osteogenic gene expression and subsequently elevating ALP activity, mineralization and new bone formation. The therapeutic effects of OA on increasing BMD in osteoporosis and on bone regeneration in fracture repair, both in mice and in humans, should be further investigated.

## References

[1] Chen C.H., Lo W.L., Liu Y.C., Chen C.Y. (2006). Chemical and cytotoxic constituents from the leaves of *Cinnamomum kotoense*. J. Nat. Prod..

[2] Wang H.M., Cheng K.C., Lin C.J., Hsu S.W., Fang W.C., Hsu T.F., Chiu C.C., Chang H.W., Hsu C.H., Lee A.Y. (2010). Obtusilactone A and (-)-sesamin induce apoptosis in human lung cancer cells by inhibiting mitochondrial Lon protease and activating DNA damage checkpoints. Cancer Sci..

[3] Cheng K.C., Hsueh M.C., Chang H.C., Lee A.Y., Wang H.M., Chen C.Y. (2010). Antioxidants from the leaves of *Cinnamomum kotoense*. Nat. Prod. Commun..

[4] Potier E., Noailly J., Ito K. (2010). Directing bone marrow-derived stromal cell function with mechanics. J. Biomech..

[5] Rosset P., Deschaseaux F., Layrolle P. (2014). Cell therapy for bone repair. Orthop. Traumatol. Surg. Res..

[6] Ma J., Both S.K., Yang F., Cui F.Z., Pan J., Meijer G.J., Jansen J.A., van den Beucken J.J. (2014). Concise review: Cell-based strategies in bone tissue engineering and regenerative medicine. Stem Cells Transl. Med..

[7] Cancedda R., Bianchi G., Derubeis A., Quarto R. (2003). Cell therapy for bone disease: A review of current status. Stem Cells.

[8] Ramamoorthi G., Sivalingam N. (2014). Molecular mechanism of TGF-β signaling pathway in colon carcinogenesis and status of curcumin as chemopreventive strategy. Tumour Biol..

[9] Choron R.L., Chang S., Khan S., Villalobos M.A., Zhang P., Carpenter J.P., Tulenko T.N., Liu Y. (2015). Paclitaxel impairs adipose stem cell proliferation and differentiation. J. Surg. Res..

[10] Bosukonda A., Carlson W.D. (2017). Harnessing the BMP signaling pathway to control the formation of cancer stem cells by effects on epithelial-to-mesenchymal transition. Biochem. Soc. Trans..

[11] Rogers M.B., Shah T.A., Shaikh N.N. (2015). Turning Bone Morphogenetic Protein 2 (BMP2) on and off in Mesenchymal Cells. J. Cell. Biochem..

[12] Hong G., Zhou L., Shi X., He W., Wang H., Wei Q., Chen P., Qi L., Tickner J., Lin L. (2017). Bajijiasu Abrogates Osteoclast Differentiation via the Suppression of RANKL Signaling Pathways through NF-κB and NFAT. Int. J. Mol. Sci..

[13] Deepak V., Kasonga A., Kruger M.C., Coetzee M. (2016). Carvacrol Inhibits Osteoclastogenesis and Negatively Regulates the Survival of Mature Osteoclasts. Biol. Pharm. Bull..

[14] Vaughan T., Pasco J.A., Kotowicz M.A., Nicholson G.C., Morrison N.A. (2002). Alleles of RUNX2/CBFA1 gene are associated with differences in bone mineral density and risk of fracture. J. Bone Miner. Res..

[15] Doecke J.D., Day C.J., Stephens A.S., Carter S.L., van Daal A., Kotowicz M.A., Nicholson G.C., Morrison N.A. (2006). Association of functionally different RUNX2 P2 promoter alleles with BMD. J. Bone Miner. Res..

[16] Osyczka A.M., Diefenderfer D.L., Bhargave G., Leboy P.S. (2004). Different effects of BMP-2 on marrow stromal cells from human and rat bone. Cells Tissues Organs.

[17] Kaewsrichan J., Wongwitwichot P., Chandarajoti K., Chua K.H., Ruszymah B.H. (2011). Sequential induction of marrow stromal cells by FGF2 and BMP2 improves their growth and differentiation potential in vivo. Arch. Oral Biol..

[18] Chen G., Deng C., Li Y.P. (2012). TGF-β and BMP signaling in osteoblast differentiation and bone formation. Int. J. Biol. Sci..

[19] Tasli P.N., Aydin S., Yalvac M.E., Sahin F. (2014). Bmp 2 and bmp 7 induce odonto- and osteogenesis of human tooth germ stem cells. Appl. Biochem. Biotechnol..

[20] Kim S.Y., Son W.S., Park M.C., Kim C.M., Cha B.H., Yoon K.J., Lee S.H., Park S.G. (2013). ARS-interacting multi-functional protein 1 induces proliferation of human bone marrow-derived mesenchymal stem cells by accumulation of β-catenin via fibroblast growth factor receptor 2-mediated activation of Akt. Stem Cells Dev..

[21] Wang C.Z., Fu Y.C., Jian S.C., Wang Y.H., Liu P.L., Ho M.L., Wang C.K. (2014). Synthesis and characterization of cationic polymeric nanoparticles as simvastatin carriers for enhancing the osteogenesis of bone marrow mesenchymal stem cells. J. Colloid Interface Sci..

[22] Chang C.H., Wang C.Z., Chang J.K., Hsu C.Y., Ho M.L. (2014). The susceptive alendronate-treatment timing and dosage for osteogenesis enhancement in human bone marrow-derived stem cells. PLoS ONE.

